# Aging Adventure Athletes Assess Achievements and Alter Aspirations to Maintain Self-Esteem

**DOI:** 10.3389/fpsyg.2018.00225

**Published:** 2018-02-28

**Authors:** Ralf C. Buckley

**Affiliations:** International Chair in Ecotourism Research, Griffith University, Gold Coast, QLD, Australia

**Keywords:** health, extreme, outdoor, nature, rafting, kayaking, participant, observation

## Abstract

Achievements and capabilities influence the self-esteem of skilled adventure athletes. Self-esteem affects individual mental health. Aging commonly reduces adventure capabilities. To avoid loss in self-esteem, aging adventure athletes are forced to adjust their aspirations. Here, I examine this process using participant observation, ethnographic and autoethnographic approaches. The qualitative data for this analysis are derived from 60 years’ experience in outdoor adventure activities, and ∼30,000 person-hours of participant observation. I argue that individuals assess their own capabilities against a set of specific feats. For some activities, successful completion of a specific feat is known as nailing it. The selection of these feats depends on factors such as activity and geographic location, as well as individual experience and peer comparisons. I examine the detailed process using a single feat repeated over a period of decades, the bubble-line kayak run through Lava Falls on the Grand Canyon of the Colorado River. I compare other examples of nail or fail to construct a general framework for self-esteem in aging adventure athletes, with both physical and psychological feedback loops. I also identify two key thresholds, as aging adventure athletes recognize their declining skills. These may apply to aging more broadly, beyond outdoor adventure.

## Introduction

If your self-esteem relies on your adventure abilities, what happens when you get old? I argue here that aging adventure athletes: measure their skills against past accomplishments; aim to maintain their capabilities as long as they can; are reluctant to acknowledge when they can’t; and are eventually compelled to adopt a different approach to adventure, accepting that they now need assistance, but can still contribute experience. Here, I analyze these processes, using an autoethnographic approach.

Self-esteem reflects self-perceived achievements in relation to self-defined aspirations (James, 1890/1983; Eromo and Levy, 2017). Different individuals focus on achievements in different aspects of their lives, social as well as physical, and give different weights to the opinions of others (Du et al., 2017; Stephenson et al., 2017; Strandell, 2017). These differences may be mediated by gender, culture, and worldview (Chen et al., 2016; Ciccolo et al., 2016). The pursuit of self-esteem can incur costs as well as benefits (Crocker and Park, 2004).

There is an extensive academic literature on self-esteem and its role in human health (Hagger et al., 2010, 2016; Chen et al., 2017; Johnson and Rasouli, 2017). One major component of this literature relates to the links between self-esteem and aging (Orth and Robins, 2014; Orth et al., 2015; Steptoe et al., 2015; Scheidt, 2016; Wooden and Li, 2016; Wheaton, 2017). Another component addresses the links between self-esteem and professional skills (Little et al., 2015; Yu et al., 2015). In general, all professions involve acquired skills. For some professions, the emphasis is on skilled thought, ideas, and knowledge; for some, on skilled action or performance; and for many, both knowledge and skilled action are necessary.

Most professional skills eventually decline, as practitioners age (Brough et al., 2011). The age at which this decline commences, and the rate of decline, differ between professions and between individuals (Erickson et al., 2014; O’Donovan et al., 2017). In general, physical skills decline at an earlier age than mental skills (DiPietro et al., 2017). However, physical exercise can maintain mental proficiency (Leckie et al., 2012; Gomez-Pinilla and Hillman, 2013; Erickson et al., 2014; Sun et al., 2016; Harvey et al., 2017; Lee et al., 2017). With particular relevance for adventure activities, aging also affects attitudes to risk (Grubb et al., 2016).

For many expert practitioners, self-esteem and self-identity are strongly bound to skill, as assessed by self and peers. This applies both to paid professions (Little et al., 2015; Yu et al., 2015), and to voluntary leisure activities (Boyes, 2013; Hickman et al., 2016, 2017; Wheaton, 2017). Self-esteem can reflect current skills, past achievements (mastery), and future potential (talent). In judging the skill of any individual relative to their peers, one objective criterion is their ability to carry out specific feats that are internationally known and ranked. Recognized feats depend on profession, may differ between regions, and may evolve over time. They may include both (a) the successful completion of formal professional qualifications, and (b) a ranked range of feats, beyond any formal test. If skills are judged by peers through a set of feats achieved, then these feats typically increase to a peak at an age which depends on the profession, and then decrease subsequently.

The overall psychological perceptions of athletes engaged in high-risk adventure activities, also known as extreme sports, have been examined in detail (Monasterio et al., 2016; Arijs et al., 2017; Brymer and Mackenzie, 2017; Brymer and Schweitzer, 2017a,b; Holmborn et al., 2017; Immonen et al., 2017). There are few recent qualitative studies, however, that examine individual adventure athletes’ perceptions of aging. There are interview-based studies of: 7 New Zealand hikers and bikers aged 63–80 (Boyes, 2013); 8 Scottish rock-climbers and 7 sea-kayakers aged 65+ (Hickman et al., 2016); 10 climbers aged 65–75 (Hickman et al., 2017); and 11 British surfers aged 45–70 (Wheaton, 2017).

## Materials and Methods

### Ethics Statement

Some of the data presented here were obtained during commercial adventure tours. Assistance and sponsorship of tour operators is gratefully acknowledged. All of the research reported here, including that drawn from previous publications by the same author (Buckley, 2010, 2012, 2015a,b, 2016, 2017), as well as new research conducted specifically for the current publication, was conducted in strict compliance with the research ethics requirements of Griffith University, including adherence to the Griffith University Research Ethics Manual and the Australian National Statement on Ethical Conduct in Human Research, and approvals by the Griffith University Human Research Ethics Committee as required.

### Autoethnographic Approach

I adopt a qualitative analytical autoethnographic methodology (Anderson, 2006; Tolich, 2010; Anderson and Austin, 2012; Buckley, 2012, 2015a,[Bibr B14], 2016; Chang, 2016; Jones et al., 2016; Pace, 2016; Stahlke Wall, 2016; Winkler, 2017). Autoethnographic approaches can yield particular accuracy, precision and subtlety in the identification of emotional responses (Buckley, 2015b, 2016; Gardner et al., 2015; Morin et al., 2015).

Autoethnographies rely on records and memories of events experienced by the analyst. Records may include photographs, videos, descriptive correspondence, and notes, journals and diaries. None of these data sources, including memory, is likely to be entirely complete or accurate (Flanagan, 1954; Brown and Kulik, 1977; Talarico and Rubin, 2003; Loftus, 2017). In the current study, however, the incidents recalled are sufficiently numerous, and extend over a long enough period, 4.5 decades, to extract robust general patterns. Memories and records may include observations (Spradley, 1980) and conversations involving other individuals. In autoethnographies, these are filtered through the perceptions of the analyst. Relevant ethical considerations and protocols are summarized by Tolich (2010).

Results are reported so that no individuals other than the author are identifiable by any mechanism, including name, role, image, geotag or other digital identifier, or place/time coordinates. This follows the autoethnographic protocols proposed by Tolich (2010). Results are also reported so that no individual incident is identifiable to a specific enterprise or sponsor. All views expressed here are those of the author, have not been discussed with any sponsors, and do not necessarily reflect the opinions of sponsor organizations or their staff.

### Data Abstraction, Generalization, and Model Building

Constructing a general model for the psychological aspects of aging in adventure athletes involves multiple steps of successive abstraction. The raw data are the moment-by-moment physical and psychological details of individual adventure practice, for particular activities, at specific sites and circumstances. In particular, they include individual memories of critical incidents, specific past events that shaped individual perceptions. These memories form the lowest-tier primary data. To gain direct access to these primary data, a researcher must have been present in person at the critical incidents concerned, as protagonist or active participant.

From these moment-by-moment memories, the first level of generalization is across the events, incidents and places experienced by an individual adventure practitioner, using their memories and perceptions. That is, we can consider or enquire how individuals perceive their own psychological changes, including aging, skills, and self-esteem. These are higher-tier data than individual memories of specific critical incidents. These higher-tier data are potentially accessible through a range of different social science methodologies. Here, I adopt an autoethnographic approach, to provide particular depth and detail (Buckley, 2015b, 2016).

These higher-tier data are based on individual memories of those incidents, but they also incorporate extensive mental processing. This processing may also include other factors perceivable by the individual, such as their own physical health, and social interactions with respected peers. They may be analyzed internally by the individuals concerned, through “self-talk” or “inner speech” (Hurlburt et al., 2016; Dickens et al., 2017). Perceptions and memories may themselves be influenced by aging, and this applies in tourism and mobility research (Huber et al., 2017; Tilley et al., 2017) as well as more generally (Bradburn et al., 1987; Knäuper et al., 2016).

The second level of generalization is across the many different individual practitioners of any one adventure activity. This second level takes into account the differences in individual personalities, capabilities, expertise, experiences, age, and life history, the “leisure lifepsychle” (Buckley, 2017). The third level of generalization is across the many different adventure activities, searching for common themes and patterns as well as distinctions and differences. This third level of generalization can adopt ethnographic or autoethnographic approaches, or it could be conducted through comparisons based on published studies. Here, I adopt the former approach, since as outlined above, there are as yet too few published studies for the latter.

### Data Presentation and Compression

In building a general model through successive abstractions from raw data, information is lost at each step. This occurs through two processes. The first is through the selection of specific critical incidents from a lifetime of experience for any one individual. The second is through the loss of detail during the attempt to identify patterns, the sacrifice of depth in the pursuit of breadth. There is thus a very high degree of data compression. In building a psychological model of adventure practitioners that is general enough to be connected to psychological theory, e.g., relating to aging or self-esteem, we thus discard the practical details that are of greatest interest to the adventure practitioners themselves.

In conversation between individual adventure practitioners, it is the finest detail of events and places that is of greatest interest. They will discuss, for example: the effect of a marginally different fin size or placement, on the performance of a kiteboard in wave jumping and wave riding, respectively; the effect of tiny changes in length, plan shape, and flexibility on the effort required to steer a snowboard, at speed, on steep soft powder snow, through dense trees; the changes in shape of surfable waves between incoming and outgoing tides; the effect of aspect and past snowfalls on avalanche risk on a particular morning; the exact hull angle and reverse paddle stroke needed to stand a particular model of kayak on its stern end in an eddy; and an indefinite number of similar considerations. In such discussions, they can identify technical details, where relevant, at scales down to millimeters and milliseconds.

In constructing and presenting a general model from a large volume of qualitative raw data, authors have a responsibility to convey to readers some sense of the type and extent of the raw data, and the steps in data compression. Various options are available to convey detailed qualitative data to a reader. The most commonly adopted approach is to include direct quotations from interviewees, but that is feasible only for interview data. Another approach, less common, is to construct first-person descriptive vignettes (Buckley, 2012). The approach used here is basic autobiographical description, without quotes or vignettes.

Similarly, there are standard techniques for compression of qualitative data. The most commonly used involve coding, i.e., the classification of an indefinite volume of raw data into a finite, iteratively defined set of low-level concepts; and simultaneously, iterative assembly of these concepts into a knowledge tree, a hierarchical structure of successively high-tier constructs. These approaches, however, require data compiled in, or convertible to, a text-based format, and are hence not readily applicable to the participant autoethnographic approach used here.

Here, therefore, I present data and analysis at two scales. First, I present a single subset of data, a narrow-scale perspective from a single route through a single rapid in a single whitewater kayaking river. Second, I present a broad-scale perspective, general patterns as I perceive them through an autoethnographic lens. These are derived from 60 years of outdoor adventure activities and aging, from my earliest memories to the present day. These broad scale patterns seem to me to apply widely across activities and individuals. The limitations of these approaches, however, suggest that they should perhaps best be seen as hypotheses, testable by other adventure practitioners using a range of relevant methodologies.

The narrow-scale analysis uses a single route through a rapid in a well-known multi-day white-water raft and kayak run, the Grand Canyon of the Colorado River. The rapid is Lava Falls, and the route is known as the bubble line. Data for this analysis are derived from multiple runs, by the author and other members of raft and kayak groups, over a period of several decades (Buckley, 2010). This case study aims to convey the degree of physical and psychological detail available in the raw and processed memories of adventure practitioners.

The broad-scale framework is derived from over half a century of outdoor adventure activities, both private and commercial, in several dozen different countries. The activities include: sea-kayaking and white-water kayaking; surfing, sailboarding and kiteboarding; skiing and snowboarding; and single and multi-day hiking (Buckley, 2012, 2016). Hundreds of persons and days, and tens of thousands of person-hours of ethnographic observations were involved. Information was obtained through: active participant observation; informal and formal face-to-face conversations either individually or in groups; and follow-up conversation using spoken, written, and electronic communications. As outlined above, analysis of this information involves successive levels of abstraction: from events, to individuals, to feats, to activities, to an overall framework.

## Results

### Nailing the Line

Expert-level practice in many skilled outdoor adventure activities involves the ability to identify, and follow, a highly specific route that allows the practitioner to pass safely and successfully through a series of obstacles. Higher levels of difficulty involve: more severe obstacles; more finely defined routes; and more rapid and precise sensory perceptions, mental analyses, and performative physical responses.

In some adventure activities, including white-water kayaking, these routes are known as lines. Sometimes there may be multiple lines, either of similar or different degrees of difficulty. Sometimes there is only one survivable line, with dire consequences for any failure, delay or deviation. Identifying a line may involve advance inspection, known as scouting. Following a line in practice requires very rapid responses to short-term sensory and proprioceptive perceptions, with skilled actions aimed at matching the actual route to one’s memory of the scouted line. If scouting is not feasible, the line must be run “blind,” which may be substantially more difficult and dangerous. A person who successfully finds and follows a line is said to have nailed it.

One component in acquiring skills is to attempt a series of successively more difficult lines, either at the same or different sites, aiming to nail each one before progressing to harder lines. As individual practitioners become more skilled, they progress from locally known training sites, to nationally or internationally known sites and lines, and then to pioneering new lines. In this process they meet peers with whom they can compare capabilities. There are multiple steps in such comparisons. For some adventure activities, including white-water kayaking, there are internationally recognized degree-of-difficulty gradings.

In white-water kayaking, individual rapids are graded on a five-point scale, Class I to Class V. Class V is sometimes subdivided, or extended to a sixth level. Individual kayakers can be graded by their ability to paddle rapids of different grades. A Class V paddler is one who is confident to tackle Class V rapids. Class V, however, especially if extended to Class VI, covers a wide range: from difficult for a Class IV paddler, to extremely difficult and dangerous even for a world-class paddler. Even where gradings exist, therefore, practitioners still compare specific feats and achievements. Initially, they do this by listing sites they have visited: mountains, rivers or surf breaks. At the next stage, they list specific obstacles or runs: named descents, rapids, or waves. Finally, they consider specific named lines.

### The Lava Falls Bubble Line

The Grand Canyon of the Colorado River in Arizona, United States is classed internationally as “big-water Class III.” This means that the hydraulic features are powerful, because the river is of moderately high-volume flow; but that the individual rapids are only of moderate technical difficulty, III on a scale I-V. The Grand Canyon also has its own internal rapid-grading system, on a more finely divided 10-point scale, but that is not comparable with other rivers. Flow rates in the Grand Canyon are much higher than many steep creeks well known to kayakers, but far smaller than high-volume rivers such as the White Nile or the Congo, or monsoon flows in Himalayan rivers such as the Karnali and Sun Khosi in Nepal, or the Nu (Salween), Li (Mekong), and Yangtze rivers in China.

Demand for river trips on the Grand Canyon far exceeds supply. Supply is restricted physically by the availability of overnight camping sites, and legally by a lottery-based permit system operated and enforced by the US National Parks Service. Certain commercial raft companies have been granted grandfathered rights to run trips throughout the year, but private groups must apply repeatedly year after year, and hope for a chance to gain a permit and run a trip. Commercial raft companies are permitted to take kayakers, but generally prefer not to do so, because it reduces their client-to-guide ratio, and hence profitability. Except for commercial raft guides, therefore, any individual white-water kayaker will typically experience few opportunities, over the course of their life, to paddle the Grand Canyon.

The highest-graded rapid in the Grand Canyon is called Lava Falls, or just Lava. It is commonplace, in many white-water rivers, that individual rapids have names. For most kayakers, Lava is the crux, the most difficult rapid on this particular river. Depending on individual experience, the same kayakers may have paddled far more difficult rapids on other rivers, or they may not. Even expert river runners will generally scout Lava before running it, since it can change over time as the riverbed shifts, and water levels increase or decrease.

There are three main lines in Lava, established by the particular conformation of the rapid, and these lines also have names. Since this is a Class III rapid, expert Class V kayakers are not restricted to the named lines. For intermediate kayakers such as the author, however, the three standard lines are known as left, V-wave, and bubble-line. Each line has its own entry point into the rapid, and the kayaker must find that entry point accurately. They must then complete a specific series of boat maneuvers and associated paddle strokes, known as “moves,” to nail the line, i.e., to follow the relevant line and exit the rapid safely. If they fail, they will get “worked” or worse. As a Class III rapid, the risk of fatality is low. To kayak Lava Falls is a skilled adventure activity, but not an extreme one in the sense of Brymer and Schweitzer (2017a,b), i.e., that any mistake is likely to prove fatal.

To explain these lines, some more detailed description and terminology is required. The main obstacle in Lava Falls is a large “ledge hole” in the center of the river, near the beginning of the rapid. A ledge hole is a hydraulic feature where water pours over a shallow underwater rock ledge. This gives the water downward vertical momentum, which punches the flow through the water surface downstream of the rock ledge, forming a seam. A kayak or swimmer falling into this seam will be sucked under water. The surface water downstream of the seam flows back upstream until it is sucked down again at the seam, forming an endlessly recirculating zone from which escape is difficult. That is, the underwater flow downstream of the ledge is shaped like a barrel with its axis across the river, its diameter reaching from surface to riverbed, rotating so the water flow is downstream on the riverbed and upstream at the surface. Similar features, also known as pourovers, occur wherever a river runs over shallow underwater rocks. The intensity of the seam, and the plan-shape of the recirculating zone, depend on river dynamics and rock shape. Some are escapable at the ends of the seam, whereas some are endless traps. The Lava Falls ledge hole is long and straight, making it difficult to escape. Therefore, all lines through Lava aim first to avoid the ledge hole.

The left line, generally considered safest, takes a kayak or raft easily past the left hand end of ledge hole (left to a person looking downstream). Depending on river flow, the left line then delivers the boat into a confused maze of subsidiary waves, with no clear course. Mishaps, however, are unlikely to have severe consequences, as any swimmers will wash through the rapid well away from the main danger zones. The lines running right of the ledge hole are more stable in shape, but require greater precision and power. The water flow is compressed between the ledge hole and the right-hand river bank. This forms a series of standing breaking waves, oriented diagonally to river flow. These diagonal waves, known as laterals, meet in pairs to form V-waves. The V-waves form a wave train. The first V-wave is particularly powerful. A boat taking the V-wave line aims to hit that largest V wave with speed and momentum, so as to punch through or over it.

A kayaker will aim to hit the right lateral wave slightly to the right of the point of the V, with their boat angled slightly to the left. This is so that the wave will push the boat diagonally toward the left, away from the river-bank, as it exits the first V-wave. This is important, since below the train of V-waves is the Black Rock, which protrudes from the shoreline into the downstream tail of the rapid. The Black Rock is potentially dangerous for three reasons. First, it is sharp: it is also called the Cheese Grater. Second, at some river levels the water flows through a narrow slot between the Black Rock and the main river bank, and a kayaker or swimmer who was washed into that slot could become stuck under water, and drown. Third, immediately downstream of the Black Rock is a powerful whirlpool, which can suck a kayaker far enough under water (as the author once discovered by experiment) that no light reaches one from the surface. The right V-wave line thus requires strong commitment and no hesitation, and the readiness to roll one’s kayak upright quickly if the V-wave hurls it upside down. It does not, however, require any very precise moves. This line is the one that most kayakers aim for.

The third line, the bubble line, is a more precise line than either of the other two. It relies on a very small section of more slowly flowing water, a sweet spot, at the extreme right hand end of the ledge hole. If a kayaker can reach that small zone upright and pointing downstream, there is a brief opportunity to avoid all the major obstacles: the ledge hole, the V-wave, the Black Rock, and some additional large waves in the center of the river downstream of the ledge hole. To follow this line, the kayaker must swing left in the sweet spot at the corner of the ledge hole, and paddle hard into the center of the river, bracing for impacts as the boat washes diagonally into these lower waves. That move requires timing, power and precision. Importantly, it also requires that the kayaker reach the sweet spot accurately, and that in turn requires nerve. Some rivers and rapids, such as the North Fork of the Payette River, require an endless series of powerful turns on marginally slower-moving water. For the Lava Falls bubble line, there is only one, but it has little margin for error.

For a kayaker entering the rapid, none of the features outlined above are visible. They are all hidden below the horizon line, where the rapid falls off from the smooth flowing water upstream. To reach the sweet spot, the kayaker must follow small cues visible in that upstream water flow. In this case, the cue is that in the smooth tongue of water entering the main flow right of the ledge hole, small boils form intermittently as water is pushed to the surface. These boils look like a line of widely spaced bubbles, each growing to about 50 cm in width and then vanishing. They are not entirely consistent in position, but generally they mark the place a kayak must be if it is to pass through the sweet spot. Hence, of course, the name bubble line. The term boil line already has a more general meaning in describing white-water hydraulics, so bubble line is the preferred name for this particular run.

The bubble line requires particular boldness, because to a kayaker entering the rapid, it appears that it leads straight into the ledge hole. If the water flowed directly downstream, it would. But it does not. The tongue of water marked by the bubble line diverts sideways around the edge of the ledge hole. The bubble line marks the extreme left hand position at which a kayaker will be carried past the corner of the ledge hole. Any further left, and the kayak will be dropped into the hole, with carnage in consequence. The water flow accelerates as it runs toward and around the corner of the ledge hole, and the kayaker can look down into the pit of the ledge hole as she or he passes it. Even more nerve-wracking, at some water flows there are several small smooth waves and one small “rooster tail” wave which one must paddle through before one can see one’s position relative to the corner of the ledge hole.

If a kayaker holds course exactly, and does not lose nerve, the water flow pushes the boat sideways past the ledge hole, and immediately below the rooster tail is the sweet spot where the powerful turn must be made. That sweet spot is less than a meter across, in the midst of a rapid over 100 m wide and hundreds of meters long. It is sometimes covered in spray, and it is right next to the roaring sound of the ledge hole. It is invisible until one is in it, yet one must be ready to react the instant one hits it. If a kayaker loses nerve even slightly, paddling even 50–100 cm right of the bubble line so as to be sure of missing the ledge hole, they will miss the sweet spot, and instead be swept into the V-wave. Worse still, instead of hitting the V-wave head-on, with speed, and momentum away from the Black Rock, they will hit it sideways, more slowly, with momentum toward the Rock. The Lava Falls bubble line is thus one of the premier tests of nerve in the entire Grand Canyon. A kayaker who nails the bubble line seems to slide cleanly through the rapid in an effortless dance; but only if they nail it. If they fail, the consequences may be messy. The Lava Falls bubble line thus provides a good tool to examine the psychology of aging in white-water kayakers.

### Autoethnographic Data

I have made five descents of the Grand Canyon, over a period of decades. The first was in a raft, as a volunteer in a research study of native fish species endangered by the Glen Canyon Dam. The other four were in a kayak. My first kayak descent was as a client in a guided commercial trip. It took place during the late 1980’s, when I was in my early thirties. The trip was principally a raft trip, but there were three or four kayakers, and the company also sent a kayak guide, as required by USNPS regulations. By the time we reached Lava Falls, which is in the lower section of the Grand Canyon, I had already “swum” twice. That is, I had accidentally tipped my kayak upside down, been unable to roll it back upright, and been forced to bail out and swim to the surface for air. These swims had occurred whilst I was trying hard to surf particular waves in my kayak, and had become exhausted through multiple attempts. They were not in dangerous places, but they had caused concern for the kayak guide. I had no swims in the other three trips, but others did, some requiring rescues.

Commercial raft trips on the Grand Canyon build up client expectations and trepidation during the night’s camp above Lava Falls, through a series of games and anecdotes. These are designed to frighten the clients, so as to increase the intensity of the experience, and incidentally their respect and gratitude to their guides for navigating them safely though the rapid. It is part of the standard emotional choreography for this product. None of the kayaking clients had run the Grand Canyon before, and we asked the kayak guide for a detailed description of Lava Falls. Using wet sand at the river’s edge, he constructed a static three-dimensional representation of the ledge hole and V-wave, and told us to aim for the center of the V-wave, point directly downstream, and paddle hard. These instructions were good, because they were simple. It is hard to recall complicated instructions when fearful.

The next morning, we reached Lava Falls and landed on the right hand bank to walk downstream and scout the rapid. The ledge hole looked very unpleasant. But I knew that people had swum through it and survived. The raft guides discussed amongst themselves whether to run right or left. Most commercial trips, where guides have nothing to prove, will run left, unless either the water level makes the right line safer, or their clients specifically ask to run the V-wave. The kayakers looked carefully at the rapid, mentally matching it to the model made by the kayak guide. The instructions were simple. Stay in the middle of the main right-side tongue, and hit the V-wave straight and hard. I could do that. But as I watched the tongue, I saw the boils forming the bubble line. I watched for a while, and saw that each one slid around the extreme end of the ledge hole. Why could I not do that too? At the time, I did not know that this was a recognized line, the bubble line. Our guide, quite rightly, was not offering us a choice. His job was just to get us safely through the rapid, despite our inexperience.

I was the weakest of the kayakers, so I was left to run last. That way, the other kayakers would be waiting at the end of the rapid, in case I might need rescuing. We all launched from the muddy bank at the same time, and waited in our kayaks, hearts beating fast. The guide told us to follow each other a few minutes apart. Should I crash the V-wave, or try to follow the boils? I had a few minutes to decide. They went fast. Either way, I should start by paddling back upstream a little, to get safely on to the tongue of water. I did so, still undecided. Then a boil broke to the surface a little to my left. I can’t remember what I thought, only what I did. I paddled to the boil, and waited. At first the flow seemed slow. Then it picked up speed. As the ledge hole came into view, it seemed that I would surely fall into it. I could see right into its maw. But I slid past, exactly at the point to take a giant paddle stroke hard left, and successfully through the rest of the rapid. As my heart rate slowed slightly, the guide paddled over, asking how I had ended up so much further left than the others. I explained. He said nothing, but shook his head slowly.

My second Grand Canyon trip was two decades later in 2008, when I was about 54. It was just after the USNPS changed the permit system for private trips, from a queue to a lottery. The queue had reached >20 years. In the changeover, they had to be sure not to disadvantage people already in the queue, since arguably those people had legal rights. They adopted a system where they gave people extra places in the lottery, depending how many years they had been in the queue. In addition, they offered that if individual trip leaders cared to combine to a single group, they could combine their lottery points. Using this approach, I found myself in a mixed private group, led by a very experienced Grand Canyon river runner. The group included young wounded US war veterans, learning to kayak under a rehabilitation program known as Wounded Warriors. They were injured and inexperienced, but fearless. They had their own kayak guides, so I was a supernumerary, left to paddle at my own pace, and occasionally lend a hand at a rescue. At Lava Falls I ran the bubble line, and once again, it went smoothly. Nobody else chose that line, and I did not suggest it.

My third Grand Canyon trip was in 2012 at age 58, on my own permit. That is, I was the one who won a place in the lottery, became the trip leader in the eyes of USNPS, and had the right to invite other participants, but the responsibility of organizing the logistics. Fortunately, I was able to invite several very experienced rafters and kayakers, who took over as leaders as soon as we were on the river. It all went well. I borrowed a kayak with plenty of leg room, and in the flat water sections, I drifted with my legs on the front deck. This was to ease the pressure on my aging spine, injured in accidents long previously. Water flow was relatively low, and Lava Falls looked less intimidating. I went first, whilst everyone else was still standing on the river bank, taking photos and videos. I ran the bubble line, but my nerve was weaker than previous trips. Instead of sticking exactly to the bubble line, I was about 50 cm to the right of the sweet spot. I passed the ledge hole safely, but got sucked backward and sideways into a plunging wave downstream, and pushed toward the Black Rock. I was able to escape with a brace, a paddle stroke that prevents a roll, but in a video of my run, one can hear comments that I was lucky.

My fourth trip was in 2016, a last-minute invitation from a generous friend of a friend. He needed a single person to replace a drop-out in a long-organized trip, to avoid the need to recalculate the entire budget. I was 62, by far the weakest and oldest kayaker. My skills were reasonably intact, but my stamina was gone. Past injuries, unrelated to adventure activities, had taken their toll, and a couple of days before we reached Lava Falls, I found myself unable to sit in the kayak all day. So, reminding myself that I was old enough to take some liberties, I hauled my kayak onto a raft and rested, whilst the younger and stronger participants did the hard work rowing. I got back into my kayak just to run the larger rapids, such as Lava Falls. Water levels were high, and the water was a rich muddy red-brown, colored by inflow from side streams. The V-wave was a chaotic liquid sculpture, tossing and churning, and the other kayakers were raring to go. They did not care about the bubble line. The boils were harder to see in the muddy water, and they thought I was making it up. They all decided to hit the V-wave. I was tempted to do likewise. But would that be a cop-out?

Was I afraid to run the bubble line? For sure, nobody else cared. But I did. It was a measure of my own self-esteem. The bubble line needs least strength, but most nerve. I was influenced by a chance encounter a few days earlier. We had met a group of scientists working on threatened native fish species. They had big motor-powered rafts, but also a speedboat. In my very first raft trip as a research volunteer, we had used an outboard-powered inflatable to get up and down the river further upstream, but we deflated it and packed it away before we ran Lava Falls. That would not be possible with a metal speedboat. I asked the speedboat pilot, a veteran fish scientist with many runs to his credit, how the logistics worked. He said that he simply ran the boat through every rapid, Lava included. I asked him whether he ran the bubble line, and he looked at me keenly for a few seconds, eventually answering only, “Yes.” That gave me pause for thought. Certainly, it was the only option smooth enough for a speedboat. But it would need nerve. A speedboat is a lot faster and more powerful than a kayak, but it can’t roll, and it would suffer much more severe damage if it ended up in the ledge hole. If he could run a speedboat through the bubble line, I thought, surely I could paddle a kayak. So I did. And yes, it went smoothly. But I’m not sure if I would do it again.

**FIGURE 1:**
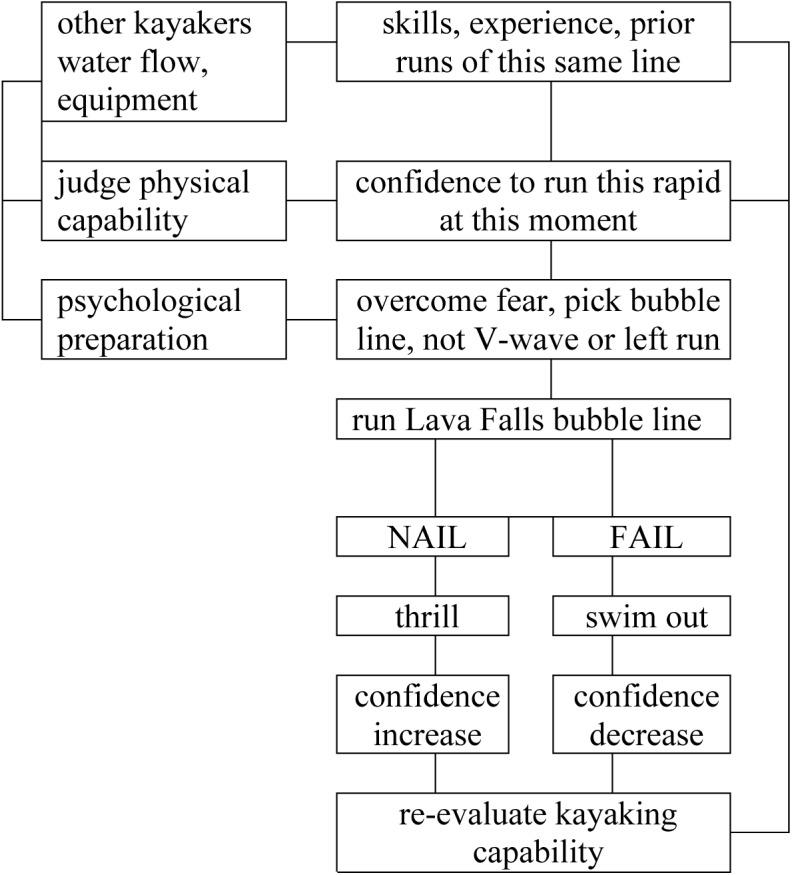
Nail or fail the Lava Falls bubble line.

What can we deduce, by comparing these recollections of runs by the same person down the same line in the same rapid, over a period of some three decades? How and why did skill, fear, thrill, and self-esteem change over that period (**Figure 1**)? Fear in anticipation, pre-event fear in the terminology of Buckley (2016), did not increase. Despite the long intervals between successive runs, one does gain a certain degree of familiarity, and hence confidence. Fear during the run itself, in-the-moment fear, was highest as I looked down into the ledge hole on the first run. Thrill was high in each case, and my recollections are not precise enough to distinguish confidently which was highest, though I think probably the first. Skill was probably highest in the second, when I had gained more experience than for the first, but had not suffered such a noticeable physical decline as during the third and fourth.

And what about self-esteem? I am glad, and relieved, that I ran the line successfully each time. I think that my self-esteem would have suffered if I had decided not to attempt it. Is this foolish? Probably. Indeed, perhaps the decision to pick the bubble line was at least partly due to pride. On the first trip, perhaps the bubble line was a way to compensate for my swims, to show that I was not incompetent. On the second, I seem to recall a feeling of ownership, that the bubble line was “my” run through Lava. On the third and fourth, when I was undeniably already old, I was testing myself against my own self-perceptions. But my self-esteem did not suffer from electing to ride on the raft during the latter part of the fourth trip. I had recognized by then that I was growing old, and had no choice but to lower my aspirations. Besides, it is not a difficult line, in global kayaking terms. It was a bucket-list item before my first trip, but after that, it is not a measure of lifetime achievement. It is not even the most difficult line in the Grand Canyon. There are other rapids with larger waves, exploding waves, rocks in the center of the river, and powerful seams. Even so, however, it provided me with a benchmark, something that showed me that despite advancing age and the many things I could no longer do, at least there were some that are still within my capabilities.

### Additional Incidents

There are other autoethnographic incidents that can be compared, to illuminate findings from the Lava Falls bubble line, and illustrate the breadth of data available. In a number of raft and kayak first descents in China many decades ago, when I was in my forties, I was the lead or probe kayaker. This is the person responsible for paddling ahead into an unknown river, judging the obstacles, and signaling back to the other boats. Some of these rivers included difficult rapids, which I ran successfully (Van Beek, 1998). These trips took place over an extended period of years, and I became older. Eventually, in my mid-fifties, I realized that I need no longer try to run every rapid or take responsibility for decisions, because we had a younger and much more competent kayaker in the group. His skills and judgment were much more reliable than my own, so my best strategy was to follow his instructions. It was both a disappointment and a relief, but it was a memorable moment, a shift in attitude. I can recall the exact place and instant when I reached this realization.

Years later again, in 2017, I took part in a multi-day raft trip on an easy, commercially run section of river in the eastern Tibetan Plateau. I was kayaking, and there was one other kayaker. She was much more competent than I, and paddled with me in a casual and friendly way. It took me some time to realize that the trip leader, much younger but much more competent than myself, had detailed her to keep an eye on me. He had not told me so explicitly, in case I might be disgruntled. It was kindly done, and allowed me to keep my self-esteem largely intact, though I could see that my skills had declined greatly from past years.

Tests of past skills do not always work out so well. In decades past, I was a heavily addicted sailboarder, and I waited for those special days when a big southerly storm would sweep in to a particular river-mouth point break. These conditions, which might happen several days in a row or not at all for years on end, brought strong south-easterly winds and mast-high or larger swells. The river mouth provided just enough shelter to get out through the breaking waves, and once well out to sea, one could pick up the green swells far out in the bay, and ride them back toward shore. As each swell neared the point, it steepened and broke, and one could turn the sailboard downwind, and ride the breaking wave like a surfer, eventually pulling over the back of the wave, just before it closed out in a flurry of unrideable foam. It took skill, strength and balance, and there was little room for error, but it was extremely addictive.

There is another site not far away, where a larger river runs out between two big rock walls, known as training walls because they train the outgoing tidal flow into a fast-flowing jet. On the biggest days of all, I would carry my sailboard into the outgoing flow, murky and sharky, and wait to be washed out through the heads of the training walls, where a big swell would break well out to sea. Once beyond the reach of the walls, there would be enough wind to raise the sail and ride the breaking waves, taking care not to be washed far out to sea, or into a rocky headland. The last time I did this, however, is many years ago.

Over the past decade, since kiteboards became widely available, my sailboard has got little use. When a moderately sizeable southerly swell rolled in during October 2017, however, I recalled past days, rigged it up, and sailed out from the (smaller) river mouth. Unlike the sailboard fleets of yore, only two other sailboarders were out there. But I found myself lacking in strength, fitness, and skill. Instead of sailing and surfing back and forth all day, I found myself worn out after only a few runs, none of them spectacular. I made an elementary mistake, and was caught in a zone with breaking waves and no wind, and washed ashore. Because of incoming tides and a sand dredge operating in the river estuary, it took me over an hour to walk back with all my gear, along the road. My aspirations were still based on memories, but my capabilities had definitely dropped. It was a rude reminder of aging.

All the data outlined above are autoethnographic. It took 1750 words to describe the Lava Falls bubble line, and a further 2000 words to describe just four runs through that line by a single kayaker, even at a basic descriptive level. Each run took a couple of minutes at most. To construct a general model, I have relied on hundreds or perhaps thousands of analogous memories, across many days of kayaking, sea-kayaking, skiing, snowboarding, surfing, sailboarding, kiteboarding and other adventure activities. Many of these memories include other participants, who watched me as I watched them, and later discussed their past experiences, skills and confidence, capabilities and feelings, in various degrees of detail. To move to a more generalized model, I rely on this much broader bank of memories, as below.

### Ethnography of Aging in Skilled Adventure Athletes

**FIGURE 2:**
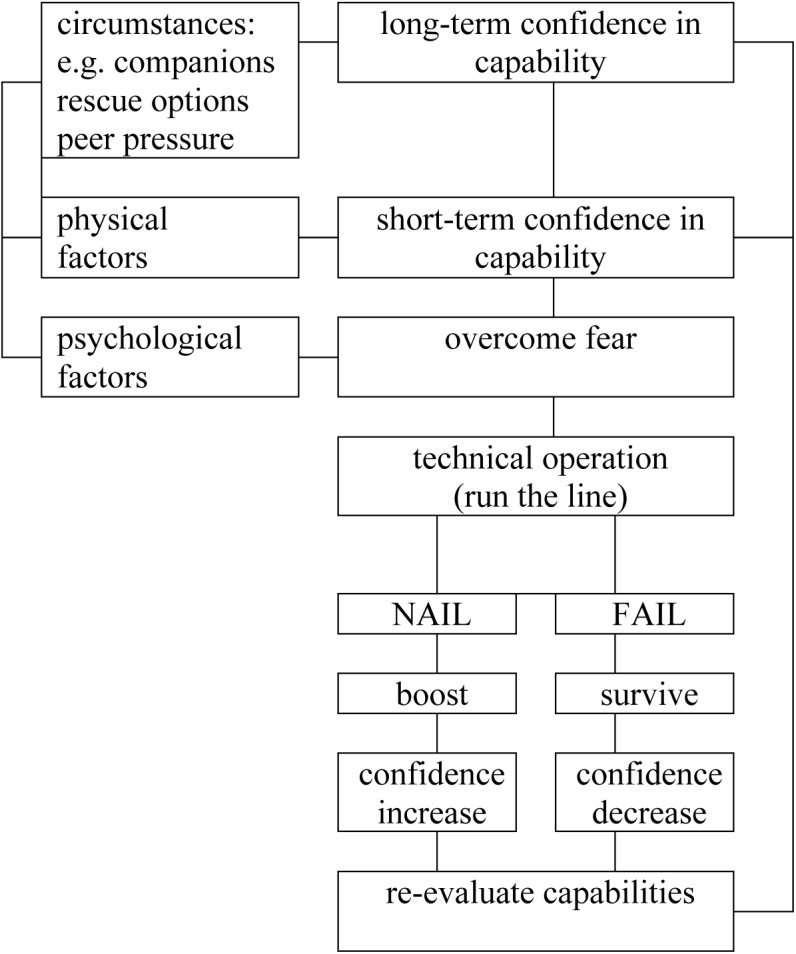
A generalized nail-or-fail model for aging adventure athletes.

In this section, I present my perceptions of general patterns associated with aging and self-esteem in skilled adventure athletes. As noted earlier, these perceptions are perhaps best treated as one person’s hypotheses, available for future testing, rather than a definitive statement. I am confident that they apply to myself, and also that they apply in general terms to many others with whom I have spent time in adventure activities. I cannot be confident, however, that they apply to individuals whom I do not know in person, or those who engage in different adventure activities, or to adventure athletes in general. With those caveats, here are my findings. Components are summarized in **Figure 2**, a generalization from **Figure 1**.

For skilled outdoor adventure athletes, the physical effects and psychological recognition of aging includes numerous subsidiary components. An individual’s reserve of strength declines with age; their reaction time slows; and their flexibility decreases. Their ability to foresee potential mishaps and plan reactions, however, may improve.

When adventure athletes are young, their skills and aspirations increase concomitantly. Initially, both are low. Typically, there may then be a period of rapid learning, when skills may exceed aspirations. As their knowledge of opportunities and peer achievements increases, their aspirations also increase. As they get older, their aspirations remain high, but their skills start to decline, and their susceptibility to injury increases. Once practitioners start to experience more mishaps and injuries, there is then a lag before they realize that they are past their prime and need to reduce their remaining aspirations. This lag, and the associated psychological adjustment, is strongly associated with self-esteem.

As the effects of aging first begin to make themselves felt, individual adventure athletes generally maintain high aspirations, in order to maintain self-esteem. During this period, their peers may assume that their skills remain adequate. The decline in skills may be hidden for a while, as practitioners can compensate to some degree, by buying better equipment and safety gear. This in itself is not an obvious indicator of decline, since the overall culture of safety is increasing for younger generations.

The decline in skills may also be hidden as individual adventure athletes begin to practice their preferred activities less frequently, because of other priorities and commitments. Even if they can see clearly that their capabilities are reduced, they may ascribe this to reduced fitness and less practice, which they may see as reversible. They may take longer to learn new skills, but attribute this to lack of practice. Overall, they expect to be able to perform as previously, but their actual physical capability has decreased. This increases the probability of misadventure and mishap. It may be these mishaps that finally force the individual to overcome this period of denial.

After this period of denial, the individuals concerned, and their peers, recognize that their skills are declining. Initially, this leads to a fall in their self-esteem. Eventually, however, they recognize that the same decline also applies for their peers, and is an unavoidable aspect of aging. With that realization, they accept it and adjust their expectations accordingly. They still wish, however, that they still had their former skills. They still struggle to maintain those skills, and they still continually measure their remaining capabilities, and attempt to minimize the decline.

During the period when individual adventure athletes recognize that their skills have begun to decline, they may also experience increasing uncertainty in their own evaluation of their own capabilities. They recognize that they are becoming slower and weaker, more easily tired, and more susceptible to heat and cold. They have more accumulated injuries and illnesses, and lower reserves of stamina. They learn to leave a safety margin when deciding how much to do in a day or a session, and they learn that they must plan exit and rescue strategies for multi-day trips. As all these factors reduce their overall capability, aging adventure athletes should logically reduce their aspirations accordingly.

They feel concern, however, that lowered aspirations might represent loss of courage, rather than an accurate reflection of reduced capability. For adventure athletes, courage is an important component of self-esteem. If it becomes difficult to distinguish whether reduced aspirations are realistic or fearful, that introduces additional uncertainty into assessment of self-esteem. This introduces a risk that individuals will attempt unrealistic feats, to prove to themselves and their peers that have not lost courage. Peer psychology has an important role to play in boosting or preventing this.

## Conclusion

### Feedback Mechanisms

**FIGURE 3:**
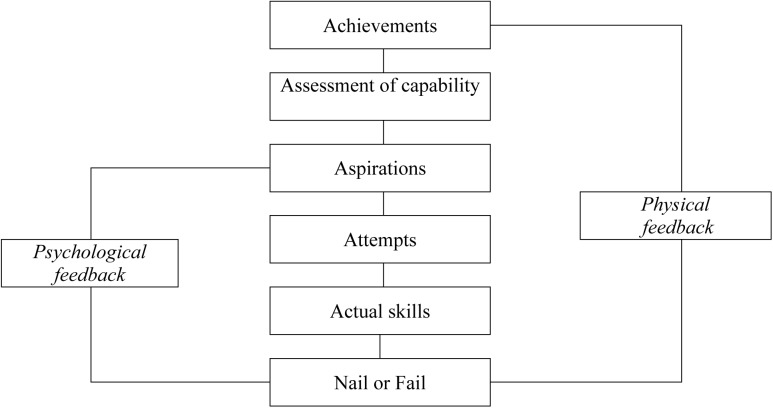
Achievements, aspirations, and assessments.

How do adventure activities influence self-esteem? Self-esteem depends on an internal comparison between achievements and aspirations (James, 1890/1983). For adventure athletes, both achievements and aspirations receive feedback via nails or fails (**Figure 3**).

### Time Dimension

I suggest that both achievements and aspirations possess a time dimension, and that this dimension has two components. The first component of the time dimension is simple: both capabilities and aspirations change over time, in line with the “leisure lifepsychle” model put forward by Buckley (2017).

The second component of the time dimension, apparently not suggested previously, is more subtle. An individual may compare his or her own achievements and aspirations either in the past, the present, or the future. The direction of view has a major effect on the outcome of the comparison; and that effect depends on age. An older individual with a lifetime of adventure achievement, for example, may recognize that they are on the downward arc of their leisure lifepsychle, but may be content with what they have done. They have no further aspirations, so their self-esteem is established by their past mastery. This self-esteem cannot be taken away, except through loss of memory.

An older individual who is still practicing adventure activities, and trying to prevent a decline in capability, may set their current aspirations with reference to past achievements. Their self-esteem depends on what they can achieve currently. As they grow older, this is likely to lead to losses in self-esteem, until the aspirations are adjusted to take account of aging. A younger individual whose skills and capabilities are still increasing is more likely to look forward, with high aspirations for future achievements. Their self-esteem is based on their own assessment of what they may achieve in future, not what they have already achieved in the past, or even what they can currently achieve in the present.

The autoethnographic accounts provided here identify two specific aging thresholds in self-assessment of achievements and aspirations, and these may apply well beyond the particular field of outdoor adventure. The first threshold occurs when an individual recognizes that there are other individuals, younger than them, who are more competent and have better judgment. During the course of the leisure lifepsychle (Buckley, 2017), younger individuals initially defer to the expertise of their elders, but eventually supplant them. The threshold identified here reflects the reverse process, when older individuals begin to defer to the judgment of their younger companions. The second threshold occurs when an aging individual recognizes that not only are they no longer a leader, but they are in fact a burden, someone who needs assistance. For self-esteem, this is a severe realization, which requires substantial mental readjustment. Perhaps these thresholds may apply to professional life histories in general.

### Basis for Comparison

Self-esteem is based on different comparisons for different individuals, depending on what they see as important (Chen et al., 2016; Du et al., 2017; Stephenson et al., 2017; Strandell, 2017). There are three main components of this: who, when and what. The first and second components depend on whether they compare only against themselves, the “personal best” school of thought; or whether they compare themselves against others, the “peer competition” approach. For example, does a kayaker compare their current rapids and runs against their own best runs in the past, or against the runs they hope to make in the future, or against the abilities of their friends currently, or against the most difficult rapids ever run and lines ever nailed by anyone anywhere? The third component is how broad a portfolio of activities and achievements they include in their comparison. Is it focussed on a single field or activity, or does it include several or many? For example, do kayakers compare themselves only on the basis of kayaking; or do they also consider other adventure activities, or other aspects of their personal and professional lives? Can a lesser achievement in one field, be offset by a broader range of fields? There is no single answer: the basis for self-esteem depends on the individual.

### Adventure, Aging, and Mental Health

Self-esteem is important to health; self-esteem of adventure athletes depends on adventure capabilities; and these capabilities decline with aging. Does this condemn aging adventure athletes to declining self-esteem, and consequently to declining mental health? In particular, could this mechanism cause faster declines in mental health for aging adventure athletes than for non-participants?

These are important questions, if we propose to adopt outdoor adventure activities as one component of mental healthcare (Clough et al., 2016; Buckley and Brough, 2017). As yet, there seems to have been no systematic tests. The evidence and arguments presented above, however, suggest that adventure has a positive overall effect on self-esteem, for two key reasons. The first reason is that nailing a line, or its equivalent in various outdoor adventure activities, provides a powerful boost to self-esteem, in a way that is not available to non-participants. As long as aging adventure athletes can continue to nail their lines, this insulates them against losses in self-esteem. The second reason is the ability to look backward as well as forward, in comparing achievements against aspirations. An aging adventure athlete who has a lifetime of nails and fails to look back upon may gain self-esteem from the past, even as they know that their future must be less athletic.

### Looking Forward

Is this field of research important? Yes. Chronic disease syndrome, various combinations of depression, dementia, diabetes and obesity, imposes costs on the economies of developed nations equal to around 10% of their current GDP (Buckley and Brough, 2017). Outdoor nature, eco and adventure therapies can reduce those costs. Outdoor exercise reduces risks of both physical and psychological ill health, and adventure activities are a key component. This is now a heavily studied field of public health and psychology, but the focus has been on physical exercise (Lee et al., 2017) and passive exposure to nature (Frumkin et al., 2017), not on outdoor adventure activities as such. That provides research opportunities for those with interests in adventure sports, tourism and recreation. Some of these research opportunities are neurobiological and physiological (Grubb et al., 2016; Sun et al., 2016; Lee et al., 2017; O’Donovan et al., 2017). Others, however, are psychological, with particular reference to self-esteem and mental health (Orth et al., 2015; Tilley et al., 2017).

What research do we need? The framework outlined above suggests a number of directions. In addition, much of the specific research cited here deserves duplication, for other adventure activities and participants. Boyes (2013), Hickman et al. (2016, 2017), and Wheaton (2017) interviewed hikers, climbers, sea-kayakers and surfers aged in their sixties and older. We need more such studies. There are corresponding groups in many adventure activities. Indeed, there are legendary characters in some activities, some still taking part in their nineties. These individuals deserve our respect, but we can also pick their brains and mine their memories. Which of their capabilities did they maintain or lose, and when, and how did it affect their self-esteem? In particular, do the two thresholds of realization identified above, where aging individuals recognize their loss of capability, apply broadly across all adventure athletes and activities? If so, might they also apply to aging more generally?

My focus here has been solely on self-esteem. There are many other parameters we might consider. Examples include: self-identity and associated concepts; mind-body interactions and performance; risk acceptance or aversion; physical skills and mental judgments; and emotional reactions to challenges and achievements. How does each of these change with aging, in different individuals? Perhaps we could analyze this through a novel research approach, which we might describe as a massively parallel autoethnography. That is, a large number of coauthors, each with professional skills in outdoor adventure, could apply autoethnographic approaches to their own experiences in parallel, and compare their results. I would gladly take part in such an approach, if others might be interested.

## Author Contributions

RB conceived, designed and conducted research, and wrote the article.

## Conflict of Interest Statement

The author declares that the research was conducted in the absence of any commercial or financial relationships that could be construed as a potential conflict of interest.
